# Cannabis compounds exhibit anti-inflammatory activity in vitro in COVID-19-related inflammation in lung epithelial cells and pro-inflammatory activity in macrophages

**DOI:** 10.1038/s41598-021-81049-2

**Published:** 2021-01-14

**Authors:** Seegehalli M. Anil, Nurit Shalev, Ajjampura C. Vinayaka, Stalin Nadarajan, Dvora Namdar, Eduard Belausov, Irit Shoval, Karthik Ananth Mani, Guy Mechrez, Hinanit Koltai

**Affiliations:** 1 Institute of Plant Science, Agriculture Research Organization, Volcani Center, 7528809 Rishon LeZion, Israel; 2 The Mina & Everard Goodman Faculty of Life Sciences, Bar-Ilan University, 5290002 Ramat Gan, Israel; 3 Institute for Postharvest and Food Science, Agriculture Research Organization, Volcani Center, 7528809 Rishon LeZion, Israel

**Keywords:** Acute inflammation, Natural products

## Abstract

*Cannabis sativa* is widely used for medical purposes and has anti-inflammatory activity. This study intended to examine the anti-inflammatory activity of cannabis on immune response markers associated with coronavirus disease 2019 (COVID-19) inflammation. An extract fraction from *C. sativa* Arbel strain (F_CBD_) substantially reduced (dose dependently) interleukin (IL)-6 and -8 levels in an alveolar epithelial (A549) cell line. F_CBD_ contained cannabidiol (CBD), cannabigerol (CBG) and tetrahydrocannabivarin (THCV), and multiple terpenes. Treatments with F_CBD_ and a F_CBD_ formulation using phytocannabinoid standards (F_CBD:std_) reduced IL-6, IL-8, *C–C Motif Chemokine Ligands* (*CCLs*) 2 and 7, and *angiotensin I converting enzyme 2* (*ACE2*) expression in the A549 cell line. Treatment with F_CBD_ induced macrophage (differentiated KG1 cell line) polarization and phagocytosis in vitro, and increased *CD36* and *type II receptor for the Fc region of IgG* (*FcγRII*) expression. F_CBD_ treatment also substantially increased *IL-6* and *IL-8* expression in macrophages. F_CBD:std_, while maintaining anti-inflammatory activity in alveolar epithelial cells, led to reduced phagocytosis and pro-inflammatory IL secretion in macrophages in comparison to F_CBD_. The phytocannabinoid formulation may show superior activity versus the cannabis-derived fraction for reduction of lung inflammation, yet there is a need of caution proposing cannabis as treatment for COVID-19.

## Introduction

Coronavirus disease 2019 (COVID-19) is an acute resolved disease following infection by SARS-CoV-2 with a mortality of ~ 3.7%. The leading cause of COVID-19 mortality is respiratory failure due to acute respiratory distress syndrome^1^. The disease progression of COVID-19 is often characterized by a two-phase immune response. A specific adaptive immune response is required during the first phase to eliminate the virus and to prevent disease progression to the more severe stage^2^. Therefore, development of strategies to increase the immune response during this first stage are critical.

The second phase is usually associated with a virally-induced cytokine storm syndrome^1,2^. The cytokine storm syndrome is characterized by elevated levels of several cytokines including interleukin (IL)-6 and IL-8, tumor necrosis factor alpha (TNFα) and C–C Motif Chemokine Ligand 2 (CCL2)^3^. Specific to the respiratory system, lung epithelial cells have been suggested to play a crucial role in the release of several pro-inflammatory cytokines including IL-6 and IL-8^4^.

*Cannabis sativa* is widely used for medical purposes. Cannabis strains produce more than 500 compounds, including phytocannabinoids, terpenes and flavonoids^5–7^. Cannabinoids have been suggested to be immune modulators and to change the balance between pro- and anti-inflammatory cytokines^8,9^. Cannabinoids can also influence macrophage activity. For example, Δ9-tetrahydrocannabivarin (THCV) inhibits nitrite production and IL-1β protein levels in lipopolysaccharide activated macrophages^10^. Further, Δ9-tetrahydrocannabinol (THC) can inhibit macrophage phagocytosis by 90%^11^. Cannabidiol (CBD) is also suggested to have anti-inflammatory effects in various conditions^12^. For example, CBD increases intracellular calcium levels in rheumatoid arthritis synovial fibroblasts and reduces the production of IL-6 and IL-8^12^. Because CBD shows anti-inflammatory activity, and is approved by the Food and Drug Administration (FDA) for the treatment of children with intractable epilepsy for seizure reduction, it has been suggested that CBD might alleviate COVID-19 related inflammation^13^. However, little is known regarding the effect of different cannabis compounds or their combinations on alveolar epithelial and immune cell inflammation.

Here, we identified cannabis compounds that exhibit anti-inflammatory activity in lung epithelial cells, yet substantially induce polarization, phagocytosis and IL expression in macrophages in vitro.

## Results

### Cannabis crude extract and fractions reduce the level of IL-8 and IL-6 in lung epithelial cell model

Inflorescence extracts of the high CBD *C. sativa* strain Arbel were used to examine cannabis activity in reducing TNFα induced inflammation in the lung epithelial cancer cell line A549.

**Figure 1:**
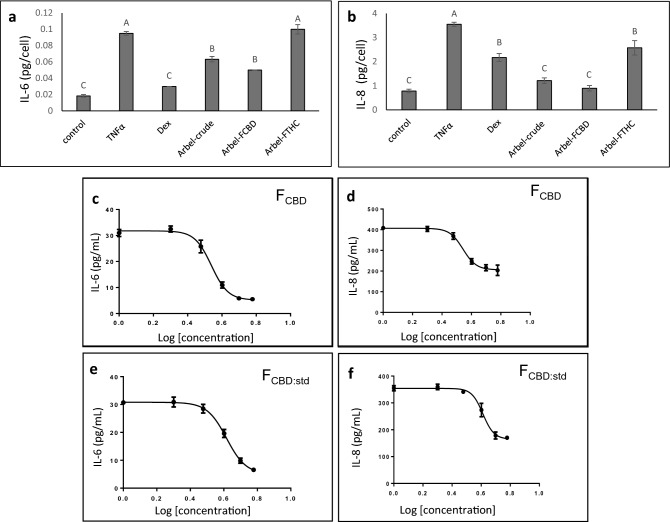
The levels of (**a**) IL-6 and (**b**) IL-8 in A549 cells treated with *C. sativa* Arbel crude and F_CBD_ and F_THC_ extract fractions. Cells were treated with 300 ng/mL TNFα and *C. sativa* extract and fractions at a concentration of 5 μg/mL for 4 h. Dexamethasone (Dex; 4 μg/mL) served as a positive control. Control (0.5% v/v methanol) treatment served as the solvent (vehicle) control; TNFα indicates TNFα+ solvent control treatment. Error bars indicate ± standard error (sem) (n = 3). Bars labeled with different letters are significantly different from all combinations of pairs by Tukey–Kramer honest significant difference test (HSD; *P* ≤ 0.05). Dose–effect curves of *C. sativa* F_CBD_ on (**c**) IL-6 and (**d**) IL-8 levels in the A549 cell line. Dose–effect curves of F_CBD:std_ (93.5% CBD+ 6.1% CBG+ 0.4% THCV) on (**e**) IL-6 and (**f**) IL-8 levels in the A549 cell line. GraphPad Prism version 6.1 was used to produce the dose–response curve and IC_50_ doses. Error bars indicate ± sem (n = 3).

The crude extract led to a substantial reduction of IL-6 and IL-8 secretion levels at 5 µg/mL (Fig. 1a,b). Subsequently, high CBD (F_CBD_) and high THC (F_THC_) fractions were examined for their anti-inflammatory activity (Fig. 1a, Supplementary Fig. S1). F_THC_ exhibited only low anti-inflammatory activity; however, F_CBD_ showed considerable activity in the reduction of IL-6 and IL-8 secretion levels from lung epithelial cells, with an IC_50_ of 3.45 and 3.49 µg/mL respectively (Fig. 1c,d). F_CBD_ reduced IL-8 levels more than dexamethasone at 4 µg/mL, and reduced IL-6 and IL-8 to levels similar to that of the crude extract (Fig. 1a,b). The crude extract and F_THC_ led to substantial cell death (61 and 42% viability, respectively), whereas F_CBD_ at 5 µg/mL was comparatively less cytotoxic (76.7% viability; Supplementary Fig. S2).

**Figure 2:**
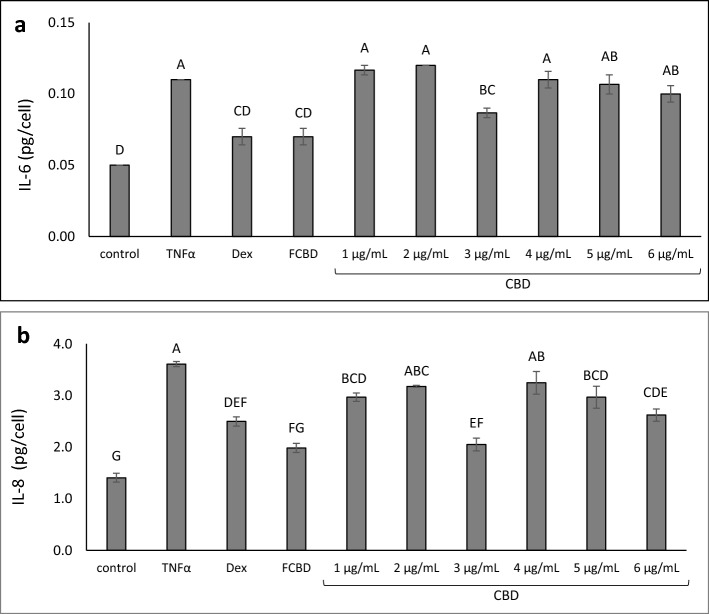
The level of (**a**) IL-6 and (**b**) IL-8 in A549 cells treated with F_CBD_ and CBD. Cells were treated with 300 ng/mL TNFα, 4.1 μg/mL F_CBD_ (FCBD) and CBD at different concentrations for 4 h. Dexamethasone (Dex; 4 μg/mL) served as a positive control. Control (0.6% v/v methanol) treatment served as the solvent (vehicle) control; TNFα is TNFα+ solvent control treatment. Error bars indicate ± sem (n = 3). Bars labeled with different letters are significantly different from all combinations of pairs according to the Tukey–Kramer HSD test (*P* ≤ 0.05).

CBD (the main phytomolecule in F_CBD_) treatment alone exhibited an inverted bell shaped activity curve, i.e., 3.0 µg/mL reduced both IL-6 and IL-8 levels, similar to F_CBD_ at 4.1 µg/mL (Fig. 2a,b). Higher or lower concentrations of CBD showed lower and/or nonsignificant reduction of IL-6 and IL-8 levels (Fig. 2a,b).

### The combination of phytocannabinoid standards at the ratios found in fraction F_CBD_ (F_CBD:std_) showed similar activity to F_CBD_ in the lung epithelial cell model

Based on high-performance liquid chromatography (HPLC) and gas chromatography–mass spectrometry analysis, F_CBD_ contains approximately 66% phytocannabinoids by total content. The phytocannabinoid assemblage included CBD (93.5%), CBG (6.1%) and a minute amount of THCV (0.4%) (Table 1; Supplementary Fig. S1). Additionally, multiple terpenes were detected in F_CBD_ (Table 1; Supplementary Fig. S3). A combination of phytocannabinoid standards at the ratios found in fraction F_CBD_ (F_CBD:std_) resulted in activity similar to that of the cannabis-derived fraction (IC_50_ of 4.1 μg/mL for IL-6 and IL-8; Fig. 1e,f).

**Table 1:** Terpene and phytocannabinoids composition in F_CBD_ as a percentage of total phytocannabinoids/terpenes and as a percentage of total compounds in extract.

Phytocannabinoids	% of phytocannabinoids	% of total
CBD	93.5	61.7
CBG	6.1	4.0
THCV	0.4	0.3

Terpenes	% of terpenes	% of total
Butylated hydroxytoluene	2.6	0.3
1,6-Dioxacyclododecane-7,12-dione	1	0.1
Guaiol	10.4	1.2
γ-Eudesmol	2.3	0.3
α-Eudesmol	5.6	0.6
Guaienol	1.3	0.2
γ-Curcumene	75.6	8.7
Other	1.2	0.1

### CB2 inverse agonist attenuated F_CBD_ and F_CBD:std_ activity in lung epithelial cell model

**Figure 3:**
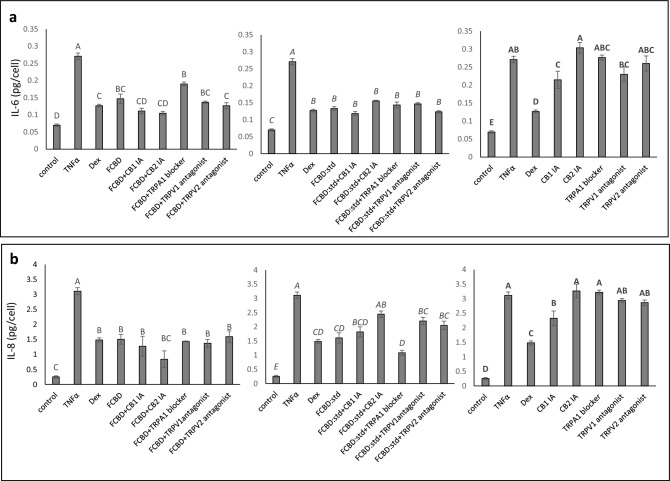
Levels of (**a**) IL-6 and (**b**) IL-8 in A549 cells treated with F_CBD_ or F_CBD:std_ with or without CB1 or CB2 inverse agonists (IA), TRPA1 blocker, or TRPV1 or TRPV2 antagonists. Cells were treated with 300 ng/mL TNFα and F_CBD_ (FCBD) and F_CBD:std_ (FCBD:std) at a concentration of 3.4 and 4.1 μg/mL, respectively, in the presence or absence of IA of CB1 (5 µM) or CB2 (5 and 7.5 µM for IL-6 and IL-8, respectively), a TRPA1 blocker (10 µM), or TRPV1 or TRPV2 antagonists (10 µM) for 4 h. Dexamethasone (Dex) served as a positive control at 4 µg/mL. Control (0.4% v/v methanol + 2% v/v dimethyl sulfoxide [DMSO]) treatment served as the solvent (vehicle) control; TNFα is TNFα+ solvent control treatment. Error bars indicate ± sem (n = 3). Bars labeled with different letters are significantly different from all combinations of pairs according to the Tukey–Kramer HSD test (*P* ≤ 0.05).

Treatment with CB1 or CB2 inverse agonists (IA), TRPA1 blocker, TRPV1 or TRPV2 receptor antagonists did not affect F_CBD_ or F_CBD:std_ activity on IL-6 secretion (Fig. 3a). Using CB2 IA and TRPV1and TRPV2 antagonists to a lesser extent, with F_CBD:std_ treatments increased IL-8 secretion in A549 cells in comparison to F_CBD:std_ alone (Fig. 3b). CB1 and CB2 IA, TRPA1 blocker or TRPV1 or TRPV2 receptor antagonists did not affect F_CBD_ activity on IL-8 secretion (Fig. 3b). Treatment with CB2 IA, TRPA1 blocker or TRPV1 or TRPV2 receptor antagonists did not affect IL-6 or IL-8 levels, except for CB1 IA that led to reduction in IL-8 and IL-6 levels (Fig. 3; Supplementary Fig. S4).

### F_CBD_ treatment lead to reduction in *CCL2*, *CCL7*, *ACE2* and *IL-7* gene expression in lung epithelial cell model

**Figure 4:**
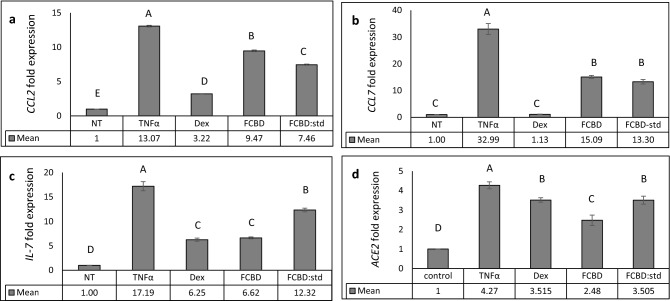
qPCR-based determination of the mRNA steady state level in A549 cell line of (**a**) *CCL2*, (**b**) *CCL7*, (**c**) *IL-7* or (**d**) *ACE2* genes, after treatment with TNFα (300 µg/mL), F_CBD_ (FCBD; 7 µg/mL) or F_CBD:std_ (FCBD:std; 7 µg/mL), or Dexamethasone (Dex; 4 µg/mL)—for 6 h relative to the control. Control (0.7% v/v methanol) treatment served as the solvent (vehicle) control; TNFα indicates TNFα+ solvent control treatment. Error bars indicate ± sem (n = 3). Bars labeled with different letters are significantly different from all combinations of pairs according to the Tukey–Kramer HSD test (HSD; *P* ≤ 0.05).

Quantitative PCR analysis demonstrated that F_CBD_ or F_CBD:std_ treatments reduced the mRNA steady state level of the pro-inflammatory cytokines *CCL2* and *CCL7* in TNFα treated A549 cells (Fig. 4a,b). However, the reduced expression of the two genes was less than those treated with dexamethasone (Fig. 4a,b). F_CBD:std_ treatment led to only a 1.3-fold reduction in the expression level of *IL-7*, whereas F_CBD_ and dexamethasone reduced *IL-7* expression substantially (2.6- and 2.7-fold, respectively; Fig. 4c). Moreover, F_CBD_, F_CBD:std_ and dexamethasone treatments reduced the expression level of *angiotensin I converting enzyme 2* (*ACE2*), F_CBD_ to a greater extent than dexamethasone or F_CBD:std_ (Fig. 4d). Expression levels of these genes were examined also at 4 h post-F_CBD_ treatment. F_CBD_ reduction of *CCL2* and *CCL7* gene expression was not apparent at 4 h, although at 4 h F_CBD_ treatment substantially reduced *ACE-2* and *IL-7* expression levels (Supplementary Fig. S5).

### F_CBD_ and F_CBD:std_ treatments induce *IL-6*, *IL-8* and *CCL2* expression in a differentiated KG1 cell line

**Figure 5:**
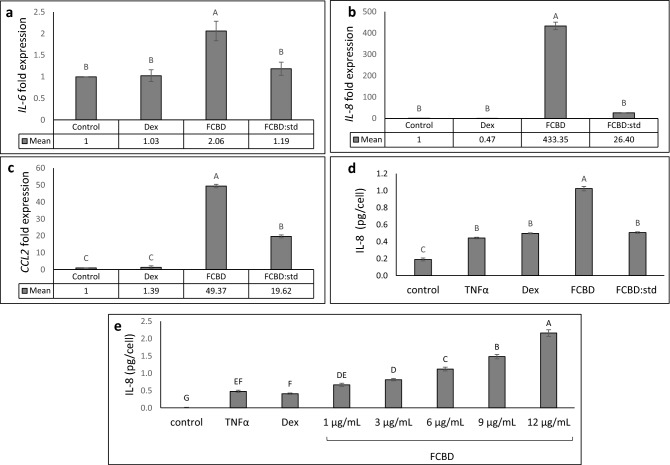
qPCR-based determination of the mRNA steady state level in the differentiated KG1 cell line of (**a**) *IL-6*, (**b**) *IL-8* or (**c**) *CCL2* after treatment with TNFα (300 µg/mL), F_CBD_ (FCBD) at 7 µg/mL, F_CBD:std_ (FCBD:std) at 7 µg/mL and dexamethasone (Dex) at 8 µg/mL for 6 h relative to control. Control (0.7% v/v methanol) treatment served as the solvent (vehicle) control. (**d**) IL-8 levels in KG1 cells treated with F_CBD_ and F_CBD-std_. Cells were treated with 300 ng/mL TNFα (and not by PMA), F_CBD_ (FCBD) or F_CBD:std_ (FCBD:std) at 10 μg/mL for 6 h. Dexamethasone (Dex; 4 μg/mL) served as a positive control. Control (1% v/v methanol) treatment served as the solvent (vehicle) control; TNFα is TNFα+ solvent control treatment. (**e**) IL-8 levels in KG1 cells treated with F_CBD_. Cells were treated with 300 ng/mL TNFα (and not by PMA) and F_CBD_ at different concentrations for 6 h. Dexamethasone (Dex; 4 μg/mL) served as a positive control. Control (1.2% v/v methanol) treatment served as the solvent (vehicle) control; TNFα indicates TNFα+ solvent control treatment. Error bars indicate ± sem (n = 3). Bars with different letters are significantly different from all combinations of pairs according to the Tukey–Kramer HSD test (*P* ≤ 0.05).

F_CBD_ treatment increased *IL-6*, *IL-8* and *CCL2* expression in phorbol-12-myristate-13-acetate (PMA)-treated (differentiated KG1 cells) macrophages by ~ 2, ~ 433- and ~ 49-fold, respectively (Fig. 5a–c). F_CBD:std_ increased *CCL2* expression by ~ 20-fold (Fig. 5c) and *IL-8* expression level by ~ 26-fold (Fig. 5b); however F_CBD:std_ did not lead to increased *IL-6* expression in macrophages (Fig. 5a). At the protein level in KG1 treated with TNFα, F_CBD_ but not F_CBD:std_ increased IL-8 secretion in macrophages (Fig. 5d). F_CBD_ activity was dose dependent (Fig. 5e). Dexamethasone (at 8 and 4 µg/mL) did not decrease expression of *IL-6*, *IL-8*, *CCL2*, or IL-8 secretion in macrophages (Fig. 5a–e, respectively).

### F_CBD_ and F_CBD:std_ attenuate macrophages polarization

**Figure 6:**
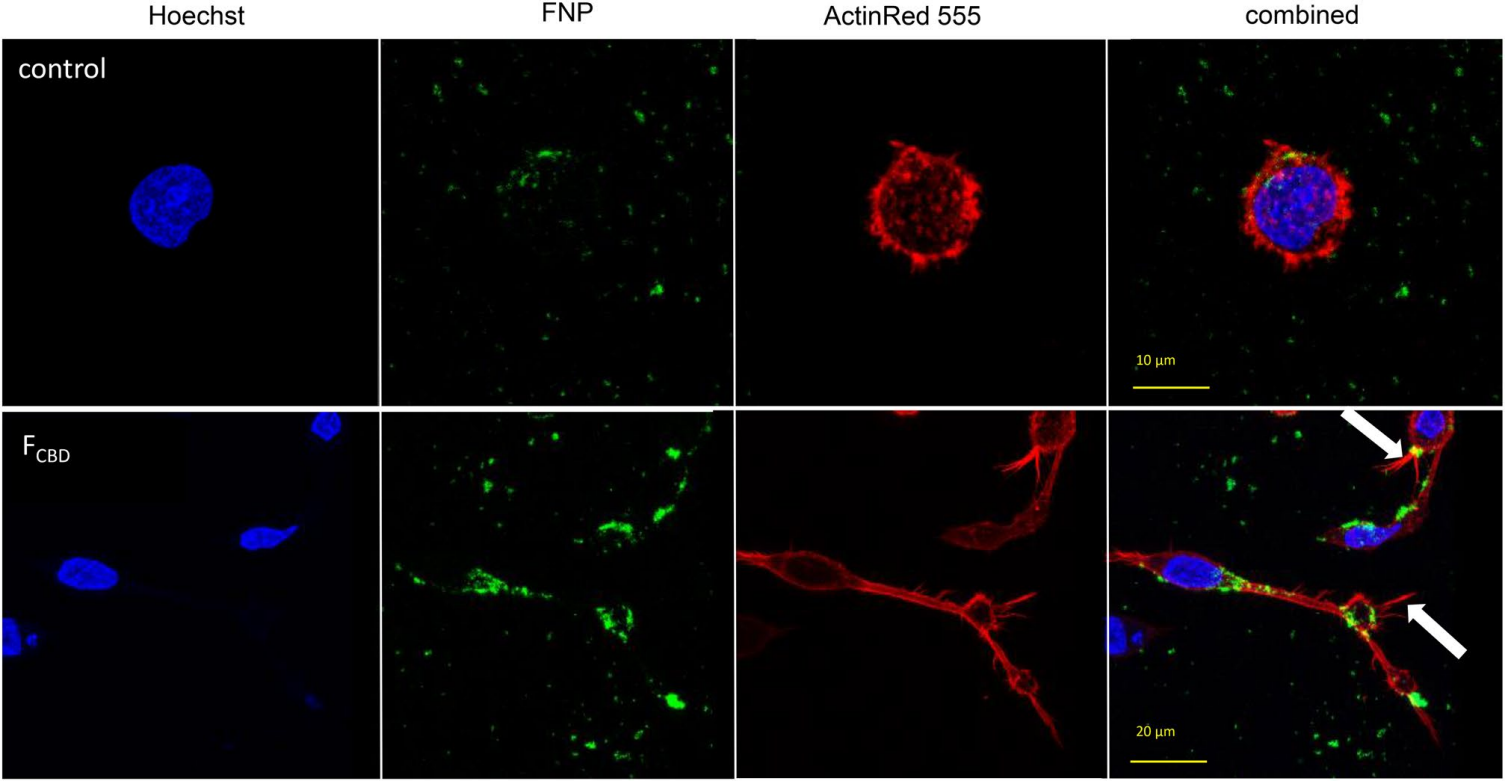
Representative examples of confocal images of macrophages following treatment with the control and F_CBD_ (7 µg/mL). Control (0.7% v/v methanol) treatment served as the solvent (vehicle) control. Cell were stained for F-actin (EasyProbes™ ActinRed 555 Stain, red stain), and nuclei (Hoechst, blue stain); n = 5, in each biological replicate multiple cells were examined (see Table 2). Membrane filopodia-like structures are marked with white arrows. FNP, fluorescent-labeled silica 50–100 nm particles.

To examine the effect of the treatments on macrophage phagocytosis we incubated PMA-treated macrophages with fluorescent-labeled silica 50–100 nm particles (FNP). In the control, most of the cells were non-polarized and featured a round structure (Table 2; Fig. 6), whereas the macrophage population treated for 16 h with F_CBD_ (7 µg/mL) consisted of ~ 48% polarized cells (Table 2). Multiple silica particles and membrane pseudopods were detected in the polarized cells (Fig. 6). Likewise, treatment of the macrophage population with F_CBD:std_ resulted in ~ 49% polarized cells (Table 2). Lower concentrations of F_CBD_ (3.5 µg/mL) led to a somewhat reduced percentage of polarized cells (~ 45%) and macrophage treatment with CBD at the equivalent concentration (4.35 µg/mL) found in F_CBD_ 7 µg/mL resulted in only ~ 18% polarized cells.

**Table 2:** Percentage of polarized cells out of the total differentiated KG1 cell population.

Treatment	% of polarized cells	Total number of cells counted in all replicates (n = 5)
Control	1.2 ± 0.83^b^	204
F_CBD_ (7 µg/mL)	48.3 ± 6.9^a^	144
F_CBD:std_ (7 µg/mL)	48.8 ± 11.3^a^	74
CBD (4.3 µg/mL)	17.9 ± 4.1^ab^	94
F_CBD_ (3.5 µg/mL)	44.9 ± 12.4^a^	70

Control (0.7% v/v methanol) treatment served as the solvent (vehicle) control. Means (n = 5 populations) labeled with different letters are significantly different from all combinations of pairs according to the Tukey–Kramer HSD test (*P* ≤ 0.05).

### F_CBD_ and F_CBD:std_ attenuate expression of phagocytosis-associated receptors

**Figure 7:**
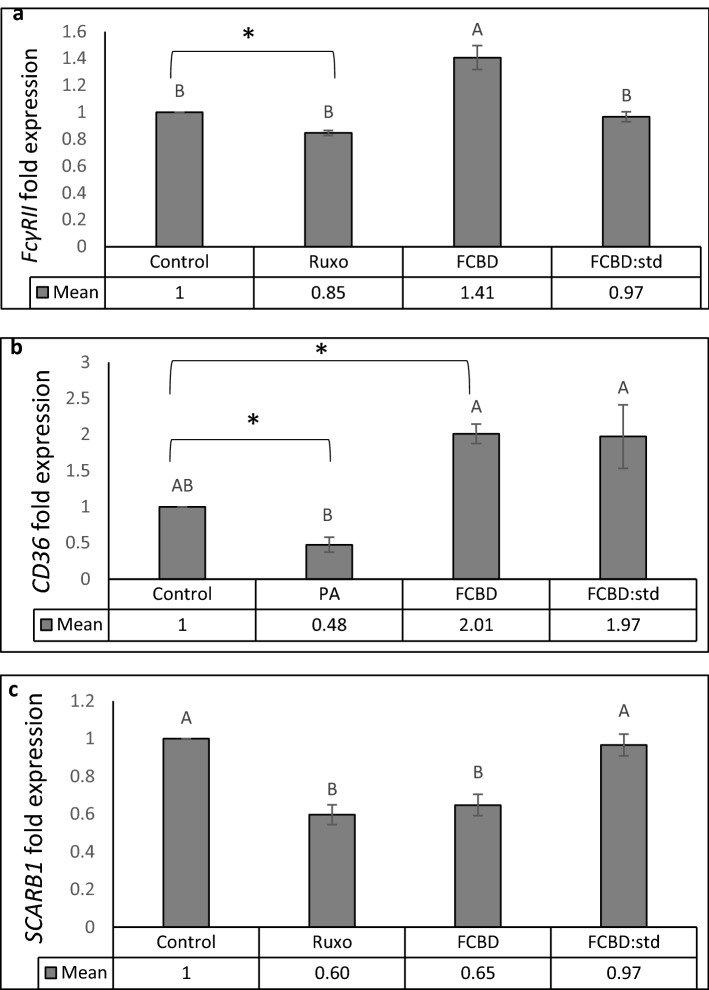
qPCR-based determination of the mRNA steady state level in the differentiated KG1 cell line. (**a**) *FcγRII*, (**b**) *CD36* or (**c**) *SCARB1* genes, after treatment with F_CBD_ (FCBD) at 7 µg/mL, F_CBD:std_ (FCBD:std) at 7 µg/mL, ruxolitinib (Ruxo) at 100 µg/mL or palmitic acid (PA) at 150 µM. In this experiment, controls (vehicle) for (**a**) and (**c**) were 0.7% v/v methanol + 2% v/v DMSO; for (**b**) 0.7% v/v methanol. Error bars indicate ± sem (n = 3). Bars labeled with different letters are significantly different from all combinations of pairs according to the Tukey–Kramer HSD test (*P* ≤ 0.05). *Indicates significantly different mean from the control based on the Student T-test (*P* ≤ 0.05).

F_CBD_ treatment, but not F_CBD:std_, increased expression of *FcγRII* and *CD36* in comparison to the vehicle control (Fig. 7a,b). Treatment with ruxolitinib which inhibits monocyte activation (described by Ahmed et al*.*^14^) reduced *FcγRII* expression (Fig. 7a), and palmitic acid (PA) reduced expression of *CD36* (Fig. 7b), in agreement with^15^. Expression of *SCARB1* was reduced by F_CBD_ and roxulitinib, but not by F_CBD:std_ (Fig. 7c).

### F_CBD_ increases silica particl internalization in macrophages

Imaging flow cytometry analysis showed that F_CBD_ increased the percentage of macrophage cells that internalized FNP (Table 3; Supplementary Fig. S6). The increase in percentage of positive cells by F_CBD_ was higher in comparison to the vehicle control also for the fluorescent-labeled silica 30–70 nm particles (ENP) and for the IgG coated, fluorescent-labeled silica 30–70 nm particles (ENPG). F_CBD:std_ and CBD treatments were less effective in increasing internalization (for FNP) or presence (ENP and ENPG) of the particles in cells in comparison to the F_CBD_ treatment (Table 3; Supplementary Fig. S6).

**Table 3:** Percentage of macrophage cells out of the control with internalized FNP silica beads, presence of ENP and ENPG silica beads analyzed using imaging flow cytometry, following treatment with F_CBD_ at 7 µg/mL, F_CBD:std_ at 7 µg/mL, CBD at 4.35 µg/mL, or the control.

Treatment	FNP	ENP	ENPG
Control	100.0^b^	100.0^a^	100.0^a^
F_CBD_	147.8 ± 13.4^a^	167.9 ± 11.2^a^	132.9 ± 30.3^a^
F_CBD:std_	99.8 ± 0.8^b^	125.3 ± 10.2^a^	116.2 ± 3.1^a^
CBD	118.85 ± 5.10^ab^	121.3 ± 24.0^a^	89.6 ± 3.9^a^

Control (0.7% v/v methanol) treatment served as the solvent (vehicle) control. Fluorescein labeled silica particles (FNP: 50–100 nm, ENP: 30–70 nm, ENPG: 30-70 nm coated with IgG). At least 4000–6000 cells for each treatment were analyzed and the distribution of cell internalization scores were plotted using Amnis IDEAS software (n = 2). Means in the same column labelled with different letters are significantly different from all combinations of pairs according to the Tukey–Kramer HSD test (*P* ≤ 0.05).

## Discussion

We have identified a CBD rich fraction (F_CBD_) from the inflorescence extract of a high CBD cannabis strain with immune-modulation activity in alveolar epithelial and macrophage cell models. F_CBD_ reduced IL-8 and IL-6 secretion in alveolar epithelial cells. IL-8 is one of the cytokines that characterizes the cytokine storm in severe COVID-19 patients; IL-6 is a prominent cytokine also involved in the cytokine storm and is secreted during the disease from alveolar epithelial cells^3^. In addition to CBD, F_CBD_ contained CBG and minute amount of THCV. The IC_50_ of a combinations of active phytocannabinoid standards (F_CBD:std_) at the relative concentrations found in F_CBD_ were similar to that of the original fraction in the alveolar epithelial cell model.

Treatment with CBD by itself led to a reduction in IL-6 and IL-8 levels in an inverse bell-shaped dose–response in alveolar epithelial cells; i.e., only 3 µg/mL was active whereas other CBD concentrations exhibited lower or no cell activity. These results are in line with an earlier publication suggesting that CBD has a bell-shaped dose–response for anti-inflammatory activity by Gallily et al.^16^. Notably, F_CBD_ (i.e., combination of CBD with CBG and THCV) led to a dose-dependent response rather than a bell-shaped dose–response. These results are in accordance with^16^, suggesting that the addition of other phytomolecules to CBD (crude cannabis extract in the case of^16^) prevented its bell-shaped dose–response. The CBD bell-shaped dose–response is associated with a narrow therapeutic window, which is difficult to use effectively in clinical therapy. Therefore, the fact that F_CBD_ has a dose-dependent response makes it better suited than CBD for patient care.

CBD is a negative allosteric modulator of CB1 signaling^17^. TRPA1 is a receptor in alveolar epithelial cells involved in the pathogenesis of several airway diseases including chronic obstructive pulmonary disease and asthma^18^. Both TRPV1 and TRPV2 interact with phytocannabinoids, including CBD, CBG and THCV^19^. Also, TRPV1, TRPV2 and TRPA1 were found to be associated with pulmonary inflammation^20^. Nevertheless, co-treatment with CB1 IA, TRPA1 blocker or TRPV1 or TRPV2 antagonist had no substantial effect on F_CBD_ and F_CBD:std_ activity. Only co-treatment with CB2 IA affected F_CBD:std_ activity on IL-8 secretion. The involvement of receptors in F_CBD_ and F_CBD:std_ activity remains to be demonstrated.

In addition to reducing IL-6 and IL-8 levels, F_CBD_ and F_CBD:std_ reduced the expression levels of *CCL2* and *CCL7* in alveolar epithelial cells by 6 h treatment. The systemic cytokine profiles detected in severe COVID-19 patients includes increased production of inflammatory chemokines such as CCL2^21^. Moreover, CCL2 and CCL7 were shown to be abundant in bronchoalveolar fluid from severe COVID-19 patients and were associated with recruitment of monocytes into the lungs^21^. Our results suggest that treatment with F_CBD_ or F_CBD:std_ may lead to reduced secretion of inflammatory cytokines associated with the disease, and possibly to a reduction of macrophage recruitment during the cytokine storm. However, dexamethasone was more effective than F_CBD_ in reducing both *CCL2* and *CCL7* expression.

IL-7 was shown to raise lymphocyte counts in septic patients with low absolute lymphocyte counts^22^ and to restore protective immunity in patients that suffer from CD4+ T cell deficiency (e.g., as in the case of HIV infection^23^). It was suggested that treatment against SARS-CoV-2 infections should also attempt to increase IL-7 levels^22^. The fact that F_CBD:std_ reduced *IL-7* expression only to a minor extent in comparison to dexamethasone or F_CBD_ suggests that using purified phytocannabinoids may have an advantage over cannabis-derived fractions for COVID-19-like inflammation.

The ACE2 receptor is a part of the dual renin-angiotensin system (RAS)^24^. ACE2 was shown to be involved with SARS-CoV-2 human infection; the ectodomain of the S protein of SARS-CoV-1 binds to the peptidase domain of ACE2 with relatively high affinity^25^. In cells of patients with severe symptoms of COVID-19, ACE2 was substantially upregulated 199-fold; this upregulation was suggested to be one of the factors leading to disruption of the RAS, as ACE2 is a part of the counteracting hypotensive axis of RAS. The increase in ACE2 and other key RAS components is predicted to elevate bradykinin levels in multiple tissues, leading to increases in vascular permeability and hypotension; the latter is highly associated with severe COVID-19 patients^26^. Indeed, a negative correlation was identified between *ACE2* gene expression and COVID-19 mortality^27^. F_CBD_ reduced the expression level of *ACE2* at 4 and 6 h post treatment. F_CBD:std_ and dexamethasone also reduced *ACE2* expression but to a lesser extent. However, the ability of F_CBD_ to reduce *ACE2* expression should be examined at both the protein and functional levels (e.g., binding of the viral protein) to fully determine the effect F_CBD_ may have on ACE2-related treatment of COVID-19 patients. In any case, such reduction of *ACE2* expression should be considered with care as the advantages and disadvantages of this reduction are disputed^24^.

In the first phase of the disease, a specific adaptive immune response is needed to eliminate the virus and to prevent disease progression to more severe stages^2^. Indeed, the dysfunction of alveolar macrophages are among the abnormal characteristics in some severe COVID-19 patients^28^, and an abundance of increased inflammatory monocyte-derived macrophages replaces tissue-resident alveolar macrophages in patients with severe disease^21^. Additionally, during SARS-CoV-1 infections that provoke a disease course similar to those seen during infection with SARS-CoV-2^21^, a marked reduction in macrophages phagocytosis activity was detected^29^. Also, phagocytosis was important in the antibody-mediated elimination of SARS-CoV-1 in a mouse model^30^.

Notably, F_CBD_ and F_CBD:std_, and CBD to a lesser extent, led to a marked increase in macrophage polarization and to cell actin remodeling that corresponds to the growth of filopodia-like membrane structures^31^. F_CBD_ reduced expression of *SCARB1*; *SCARB1* encodes SR-B1 that is a scavenger receptor (class B) and is also responsible for phagocytosis of silica particles in macrophages^32^. However, F_CBD_ treatment also led to an increase in *FcγRII* and *CD36* gene expression. Phagocytosis is initiated by the ligation of Fcγ receptors to IgG-opsonins on the target cell^33^, whereas CD36 expression in macrophages was shown to be involved with lung fibrosisin in mice^34^. Alveolar macrophages play an important role in Fc receptor-mediated responses during acute virus infections and in phagocytosis-mediated clearance of respiratory virus infections^35,36^. CD36 is an important scavenger receptor for phagocytosis of *Streptococcus pneumoniae*, a primary bacterial agent associated with pneumonia, which is down regulated by influenza^37^. Indeed, F_CBD_ led to a marked increase in the internalization of silica particles by macrophages, and in so doing, increased levels of phagocytosis.

Possibly, the increase in macrophage polarization and phagocytosis, and the upregulation of *FcγRII* and *CD36* expression in these cells following F_CBD_ treatment may facilitate phagocytosis-mediated clearance of respiratory viruses, and benefit the first phase of the immune response to SARS-CoV-2. However, it should be noted that macrophages themselves can be infected by the virus, as SARS-CoV-1 infects macrophages as a result of IgG-mediated phagocytosis that requires FcγRII receptor signaling pathways^38^. Advantages and disadvantages of increasing macrophage phagocytosis activity should be carefully considered^3,21^.

Notably, although F_CBD:std_ treatment increased macrophage polarization, it did not increase the phagocytosis-associated gene expressions, nor phagocytosis. Hence, additional active compounds in the cannabis-derived F_CBD_ and not in the phytocannabinoid standard mix that composed F_CBD:std_ are responsible for this increased gene expression and phagocytosis activity. Indeed, F_CBD_ contained multiple terpenes, some including γ-Curcumene and Guaiol at considerable percentages. The presence of terpenes in F_CBD_ may account for the differences in activity between F_CBD_ and F_CBD:std_.

During the second phase of COVID-19, pneumonia patients exhibit features of macrophage activation syndrome (MAS) in which macrophages play a major pro-inflammatory role by releasing pro-inflammatory cytokines such as IL-6, IL-8 and CCL2^3^. Moreover, subsets of macrophages in patients with COVID-19 were found to express genes associated with IL-6, whereas expression of IL-6 was again associated with severe depletion of lymphocytes from the spleen and lymph nodes^21^. Notably, F_CBD_ led to a marked increase of *IL-8* expression and IL-8 protein levels in macrophages. It also led to an increase in *IL-6* expression levels, above that induced by PMA^39^. These results suggest a substantial, in vitro, pro-inflammatory role for F_CBD_ in macrophages. However, F_CBD;std_ was less active in ILs induction, again demonstrating a notable difference between F_CBD_ and F_CBD:std_, which may originate from the presence or absence, respectively, of terpenes.

To conclude, treatment with cannabis compounds CBD, CBG and THCV may have clinical value in reducing cytokine secretion in lung epithelial cells. However, treatment with F_CBD_ containing terpenes in addition to these phytocannabinoids substantially induced macrophage phagocytosis and increased their IL levels. Yet, to confirm more specifically the pro-inflammatory effect of F_CBD_ in macrophages it is necessary to perform the same experiments on primary alveolar macrophages (e.g., from mice). Nevertheless, these results suggest a pro-inflammatory role for cannabis extract that is higher than that of the phytocannabinoid standard mix. The latter maintained anti-inflammatory activity in the alveolar epithelial cells with relatively reduced pro-inflammatory activity in macrophages. Hence, the mix of phytocannabinoids shows superior activity versus the cannabis-derived fraction. Although more studies are needed of cannabis treatment in COVID patients, there needs to be caution in proposing cannabis treatment for these patients, as is presently being suggested in the media. The increase of macrophage-secreted IL-6 and IL-8 levels by cannabis-based treatment may potentially lead to a worsening of the "cytokine storm" identified in severe COVID-19 patients. It should be stressed, in agreement with Pastor et al*.*^40^, that for now, users and healthcare personnel should avoid the use of cannabis for COVID-19 prevention or treatment.

## Material and methods

### Extract preparation

High CBD *Cannabis sativa* strain Arbel (IMC, Israel) inflorescence was extracted using ethanol as described previously^41^ and decarboxylated by heating the dried crude extract to 220 °C for 10 min. The dried decarboxylated extract was weighed, and then resuspended in absolute methanol (volume of solvent added according to the desired concentration) and filtered through a 0.45 μm syringe filter.

### Extract fractionation

A flash chromatography apparatus equipped with a diode array detector was used to fractionize the decarboxylated crude extract. An Ecoflex C-18 80 g (Flash Pure, Buchi, C-18, 50 µm spherical, max. pressure 180 psi) column was used for separation, with methanol and water as the mobile phase, as suggested by the manufacturer.

### Chemical analyses

HPLC and gas chromatograph with mass selective detector (GCMS 8860 and GC/MSD 5977B, Agilent) analysis was carried out as previously described^41^. Qualitative and quantitative analysis of the phytocannabinoids in fractions was done in comparison to the standard calibration curves obtained from dissolving standards in methanol at different concentrations from 0 to 25 µg/mL.

### Standard/material preparation and use

The cannabinoid standards at a concentration of 1 mg/mL in methanol used in this study included cannabidiol (CBD, Restek catalog no. 34011) cannabigerol (CBG, Restek catalog no. 34091) and tetrahydrocannabivarin (THCV, Restek catalog no. 34100). Inverse agonists (IA) to cannabinoid receptors type 1 (CB1) and 2 (CB2) used were Abcam products: CB1 (AM251, ab120088), CB2 (SR144528, ab146185), as was the TRPA1 blocker (HC-030031, ab120554). All IAs, as well as the TRPV1 antagonist (Abcam ab141772) and TRPV2 antagonist (Tranilast 1098/10) were dissolved in dimethyl sulfoxide (DMSO) at a concentration of 10 mM. Phorbol 12-myristate 13-acetate (PMA) (P1585; Sigma Aldrich, USA) was dissolved in DMSO at the stock concentration of 5 µg/mL. Dexamethozone (D4902; Sigma Aldrich, USA) was dissolved in methanol at the stock concentration of 1000 µg/mL. Ruxolitinib JAKAVI was dissolved in DMSO at the concentration of 5000 µg/mL, confirmed with GCMS and HPLC and was used at a final concentration of 2% (v/v). TNFα (300-01A; PeproTech, Rocky Hill, NJ, USA) was dissolved in water at the stock concentration of 100 µg/mL. (3-Aminopropyl) triethoxysilane (APTES), N-(3-Dimethylaminopropyl)-N(3-ethylcarbodiimide hydrochloride (EDC), and 5(6)- Carboxyfluorescein, 2-(4-Morpholino) ethanesulfonic acid (MES) were purchased from Sigma-Aldrich (USA). Analytical grade methanol was used at final concentration of 10% (v/v) according to the indicated concentration of the treatment. The highest methanol concentration in each experiment was used for vehicle control. Ultra-pure deionized water (MS grade) was used as received without further purification. Palmitic acid (PA, Sigma Aldrich; P0500, USA) was dissolved in methanol at the stock concentration of 0.5 mol/L and used at 150 µM.

### Cell cultures

The lung cancer cell line A549 (ATCC^®^ CCL-185™) was cultured in DMEM (01-055-1A, Biological Industries, Israel) growth media supplemented with 10% FBS, 1% glutamic acid, 1% pen-strep and plasmocin. Macrophage cell line KG1 (ATCC^®^ CCL-246™) was cultured in IMDM (01-058-1A; Biological Industries, Israel) containing 20% FBS and 1% pen-strep and plasmocin. 10 ng/mL PMA in IMDM media supplemented with 5% FBS, 1% pen-strep and plasmocin was used as stimulating environment for the differentiation of KG1 cells. Differentiated cells with typical morphology were attached to the plate surface within 1–2 days of initiation^42^.

### Determination of IL levels and cell viability

IL-6 and IL-8 levels were determined as described previously^43^ with the following modifications: A549 cells were plated at 5 × 10^4^ cells per well in DMEM complete media (400 µL) in 24-well cell culture plate. They were allowed to attach and grow at 37 °C in air and 5% CO_2_ in a humidified incubator overnight with complete DMEM, and then the media was replaced with serum free DMEM. Cell excitation was performed with 300 ng/mL TNFα. Treatments were performed with cannabis crude extract, fraction or combination of compounds together with 100 µL serum free DMEM. IL-6 and IL-8 secretion levels were analyzed after 4 or 6 h of incubation for A549 or KG1 cell lines, respectively. Supernatant samples were collected and tested using IL-6 and IL-8 ELISA kits (DY206 and DY208 respectively, R&D Systems, Minneapolis, MN, USA). Dexamethasone was used as a positive control. For cell viability, an Alamar Blue (resazurin) assay was performed on each well as described previously^43^. For dose response assays, data points were connected by non-linear regression lines of the sigmoidal dose–response relation. GraphPad Prism version 6.1 (https://www.graphpad.com/scientific-software/prism/, GraphPad Software Inc., San Diego, USA) was employed to produce dose–response curves and IC50 doses were calculated using nonlinear regression analysis.

### Salinization of silicon dioxide surfaces with APTES

To prepare the silica dispersion, 1 g of silica was added to 40 mL of methanol and stirred. Then, APTES (0.0045 mol) was slowly added to the solution. The reaction was carried out at ambient temperature for 45 min. After silanization, 50–100 nm or 30–70 nm particles were collected by centrifugation (9000 rpm, 10 min) washed 4 times with water, and dried at 35 °C under vacuum for 3 h^44^.

### Labeling of amine functionalized silica nanoparticles with 5(6)-carboxyfluorescein and IgG

Stock solutions of 1 mg of EDC were prepared separately in 1 mL of 0.1 M MES (pH 4.5–5) buffer. 100 mg of the amine functionalized silica nanoparticles were added to 600 µL of the MES buffer followed by 200 µL of the EDC. The mixture was vortexed for 10 min. Then 100 µL 5(6)-Carboxyfluorescein (1 mg/mL) only (for 50–100 nm FNP or 30–70 nm ENP nanoparticles) or 100 µL 5(6)-Carboxyfluorescein (1 mg/mL) and IgG (10 mg/mL; for 30–70 nm ENPG nanoparticles) solutions were added. The final solution was then mixed by vortex for 3 h at ambient room temperature. Subsequently, the mixture was centrifuged and rinsed with MES buffer to remove excess reactants. EDC was used as a cross-linker to chemically attach the carboxyl group of the 5(6)-Carboxyfluorescein molecule and producing an amine-reactive O-acylisourea. For the fluorescent-IgG labelled silica nanoparticles this intermediate product reacted with the amino groups of the silica nanoparticles to yield an amide bond, releasing fluorescent-IgG labelled silica nanoparticles and urea as a by-product^45^. The fluorescent labelled (FNP or ENP) or fluorescent-IgG labelled (ENPG) silica nanoparticles were then dispersed again in the MES buffer for further analysis.

### Cellular staining and confocal microscopy

Differentiated macrophages from KG1 cells (10 × 10^4^ cells/plate; plated on the bottom of a glass cell culture dish) were incubated in 500 µL of 5% FBS-IMDM media with FNP, ENP or ENPG (40 µg/mL) and incubated at 37 °C for 4 h for phagocytosis. Macrophages that underwent phagocytosis were fixed with 3.7% formaldehyde solution and permeabilized with 0.1% Triton X-100 at room temperature. Fixed cells were blocked in PBS containing 1% bovine serum albumin. Cells were incubated with EasyProbes™ ActinRed 555 Stain for actin and Hoechst for nuclear staining (AP-FP032, GC-C057 respectively; ABP Bioscience Rockville, MD, USA). Cell microscopy and image acquisition was carried out using a Leica SP8 laser scanning microscope (Leica, Wetzlar, Germany), equipped with a 405, 488 and 552 nm solid state lasers, HCX PL APO CS 10×/0.40 or HC PL APO CS 63×/1.2 water immersion objectives (Leica, Wetzlar, Germany) and Leica Application Suite X software (LASX, Leica, Wetzlar, Germany). Hoechst, 5(6)-Carboxyfluorescein and ActinRed 555 emission signals were detected with PMT and HyD (hybrid) detectors in ranges of 415–490 nm, 500–535 nm and 565–660 nm, respectively.

### Quantitative real-time PCR

Quantitative real time PCR (qPCR) was carried out as described previously^41^. Briefly, cells were treated with cannabis compounds or methanol (0.7% v/v) as vehicle control for 4 or 6 h. Cells were then harvested and total RNA was extracted. RNA was reverse-transcribed, primers were designed and PCR was performed. The expression of each target gene was normalized to the expression of *Hypoxanthine Phosphoribosyltransferase 1* (*HPRT1*) mRNA in the 2^−ΔΔCt^ and is presented as the ratio of the target gene to HPRT mRNA, expressed as 2^−ΔCt^, where Ct is the threshold cycle and ΔCt = Ct Target − Ct HPRT1. Experiments were repeated three times. The primers were:

- *ACE2* (Gene ID: 59,272) (forward) 5′-AAGCACTCACGATTGTTGGG-3′ (reverse) 5′-CACCCCAACTATCTCTCGCT -3′;
- *CCL2* (Gene ID: 6347) (forward) 5′-AAGGAGATCTGTGCTGACCC-3′ (reverse) 5′-GCTGCAGATTCTTGGGTTGT -3′;
- *IL-6* (Gene ID: 3569) (forward) 5′-GAACTCCTTCTCCACAAGCG-3′ (reverse) 5′-GAAGAGGTGAGTGGCTGTCT-3′;
- *CCL7* (Gene ID: 6354) (forward) 5′-CACCCTCCAACATGAAAGCC-3′ (reverse) 5′-GGTGGTCCTTCTGTAGCTCT -3′;
- *IL-7* (Gene ID: 3574) (forward) 5′-CTGAAAGTACACTGCTGGCG-3′ (reverse) 5′-GAGTTGCCGAGTCTGTGTTG3′;
- *FCγR2A* (Gene ID: 2212) (forward) 5′-GCC AAT TCC ACT GAT CCT GT-3′ (reverse) 5′-CCTGGGGTTCAGAGTCATGT-3′;
- *SCARB1* (Gene ID: 949) (forward) 5′-CTG TGG GTG AGA TCA TGT GG-3′ (reverse) 5′-GTT CCA CTT GTC CAC GAG GT-3′;
- *CD36* (Gene ID: 948) (forward) 5′-AGA TGC AGC CTC ATT TCC AC-3′ (reverse) 5′-TGG GTT TTC AAC TGG AGA GG-3′;
- *IL-8* (Gene ID: 3576) (forward) 5′-CAG GAA TTG AAT GGG TTT GC-3′ (reverse) 5′-AAA CCA AGG CAC AGT GGA AC-3′.

### Imaging flow cytometry

Differentiated macrophages from KG1 cells (10 × 10^5^ cells/plate; seeded on 6-well plate culture dish) were replaced with 2 mL of 5% FBS-IMDM media with FNP, ENP, or ENPG (40 µg/mL) and incubated at 37 °C for 4 h for phagocytosis. The cells were detached from the surface of the plate using a trypsin 0.25%:EDTA 0.05% solution (03-052-1A, Biological Industries, Israel) for 3 min, washed with DMEM complete media, centrifuged and transferred to 50 µL cold PBS kept on ice.

Cells were analyzed by multispectral imaging flow cytometry (ImageStream markII flow cytometer; Amnis Corp, part of EMD Millipore, Seattle, WA, USA). Fluorescence intensity of the Fluorescein labeled silica beads was measured in channel 2 of the cytometer (480 nm ex, 560 nm em). An 60× magnification with Olympus UplanFLN 60× dry objective 0.9NA was used for all samples. At least 4000 cells were collected for each sample and data were analyzed using a dedicated image analysis software (IDEAS 6.2; AmnisCorp). Cells were gated for single cells using the area and aspect ratio features, and for focused cells using the Gradient RMS feature. Cropped cells were further eliminated by plotting the cell area of the bright field image against the Centroid X feature (the number of pixels in the horizontal axis from the left corner of the image to the center of the cell mask). Cells were further gated for cells that were positive (for ENP, ENPG or FNP). Because of their larger size, only FNP beads could be further analyzed for bead internalization vs. those attached to the cell surface. This was done using the intensity feature (the sum of the background-subtracted pixel values within the masked area of the image) and max pixel (the largest value of the subtracted background pixel). FNP internalization was calculated by the internalization feature, i.e. the ratio of the intensity inside the cell to the intensity of the entire cell, mapped to a log scale. To define the internal mask for the cell, the object mask of the brightfield image was eroded by 8 pixels. Cells with an internalization score higher than 0.33 were gated as cells with internalized FNP.

### Statistical analysis

Data were processed using the JMP statistical package (https://www.jmp.com/en_us/home.html, SAS Inc, NC, USA). Comparisons between two groups were made using the Student’s T-Test. Comparisons between more than 2 groups were made with analysis of variance (ANOVA) followed by Tukey–Kramer's honest significant difference (HSD) test as post hoc. Values are shown as mean ± standard error (sem). *P* values ≤ 0.05 were considered significant.

## Supplementary Information

Supplementary Figures.

## Data Availability

The datasets generated during and/or analysed during the current study are available from the corresponding author on reasonable request.
