# Supplementary material for: Cannabis compounds exhibit anti-inflammatory activity in vitro in COVID-19-related inflammation in lung epithelial cells and pro-inflammatory activity in macrophages
Source: Sci Rep. 2021 Jan 14;11:1462. doi: 10.1038/s41598-021-81049-2 (PMC7809280; doi:10.1038/s41598-021-81049-2)
Supplement: Supplementary file 1 — Supplementary Figures. [file 41598_2021_81049_MOESM1_ESM.pdf]

Supplementary Information for:

Cannabis compounds exhibit anti-inflammatory activity *in vitro* in COVID-19-related inflammation in lung epithelial cells and pro-inflammatory activity in macrophages

Seegehalli M Anil, Nurit Shalev, Ajjampura C Vinayaka, Stalin Nadarajan, Dvory Namdar, Eduard Belausov, Irit Shoval, Karthik Ananth Mani, Guy Mechrez, Hinanit Koltai

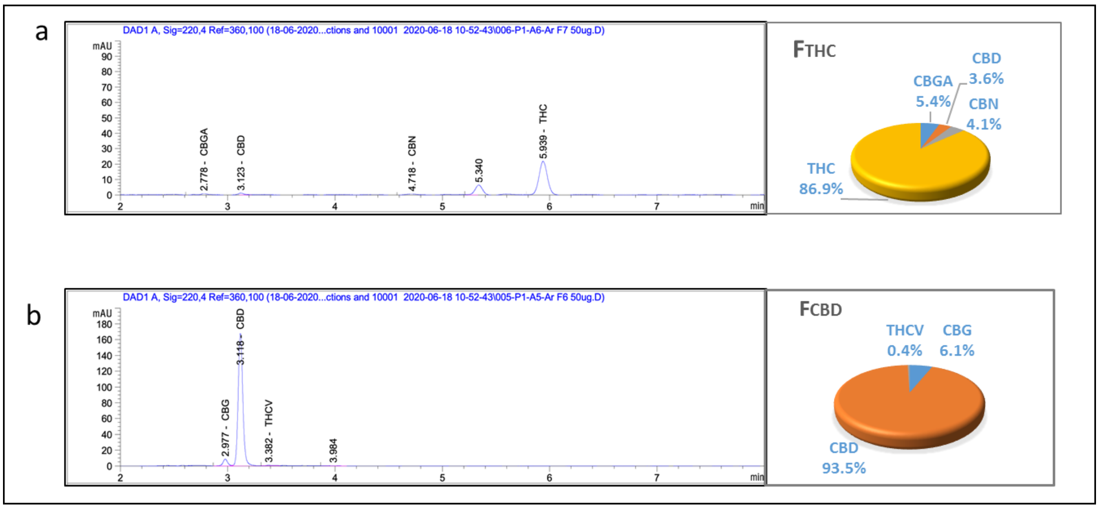

Supplementary Figure S1. HPLC profile of *C. sativa* Arbel extract fractions (a) FCBD, (b) FTHC. The phytocannabinoid composition of the extract fractions is shown as a percentage of the total phytocannabinoid content in the pie charts.

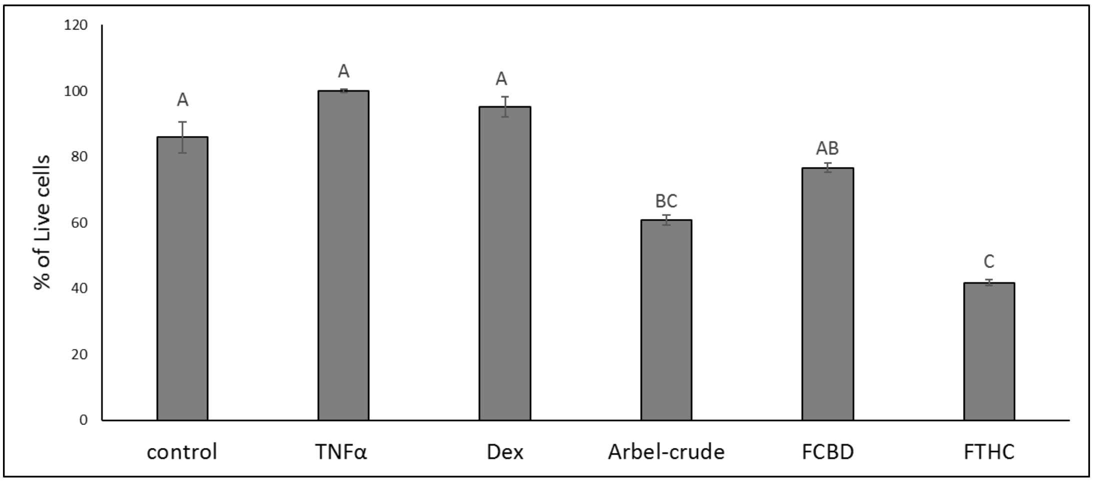

Supplementary Figure S2. Cell viability in A549 cells treated with the crude extract and *C. sativa* Arbel extract fractions FCBD and FTHC. Cells were treated with 300 ng/mL TNFα and the *C. sativa* extract or extract fractions at a concentration of 5 μg/mL for 4 h. Dexamethasone (Dex; 4 μg/mL) served as a positive control.

Control (0.5% v/v methanol) treatment served as the solvent (vehicle) control; TNFα is TNFα+solvent control treatment. Error bars indicate ± s.e.m. (n = 3). Bars with different letters are significantly different from all combinations of pairs according to the Tukey-Kramer honest significant difference test (HSD; $P \leq 0.05$).

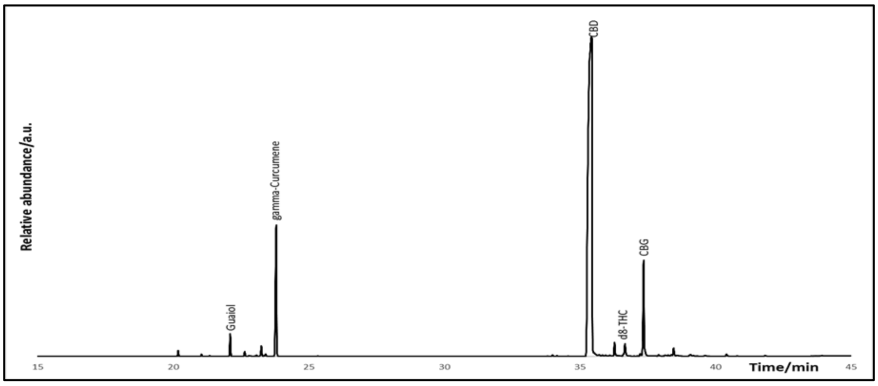

Supplementary Figure S3. Gas chromatogram of F_{CBD}, with the major peak indicating compounds labeled.

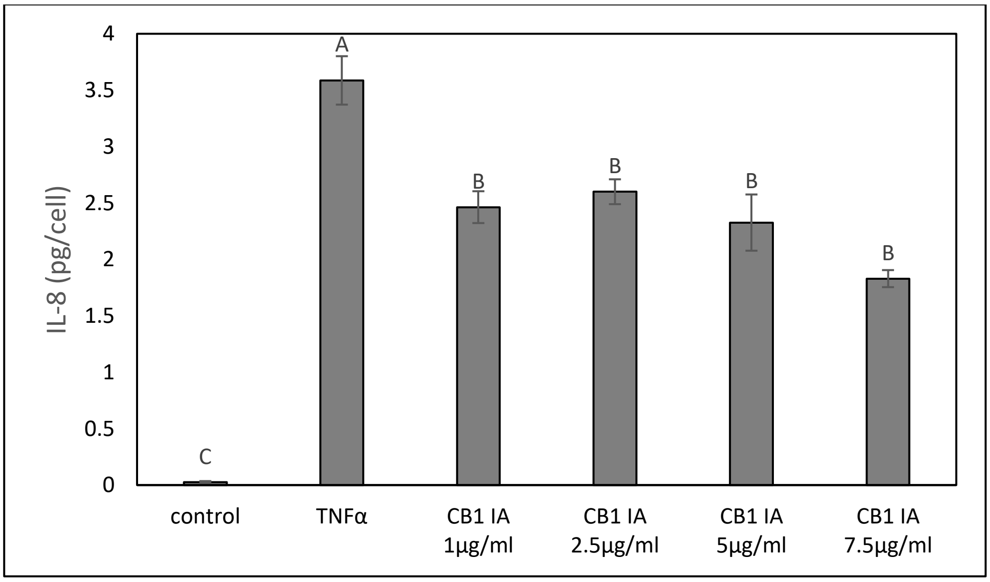

Supplementary Figure S4. Levels of IL-8 in A549 cells treated with CB1 inverse agonists (IA). Cells were treated with 300 ng/mL TNFα and CB1 IA at different concentrations for 4 h. Control (0.4% v/v methanol + 2% v/v dimethyl sulfoxide [DMSO]) treatment served as the solvent (vehicle) control; TNFα is TNFα+ solvent control treatment. Error bars indicate ± s.e.m. (n = 3). Bars labeled with different letters are significantly different from all combinations of pairs according to the Tukey-Kramer HSD test ($P \leq 0.05$).

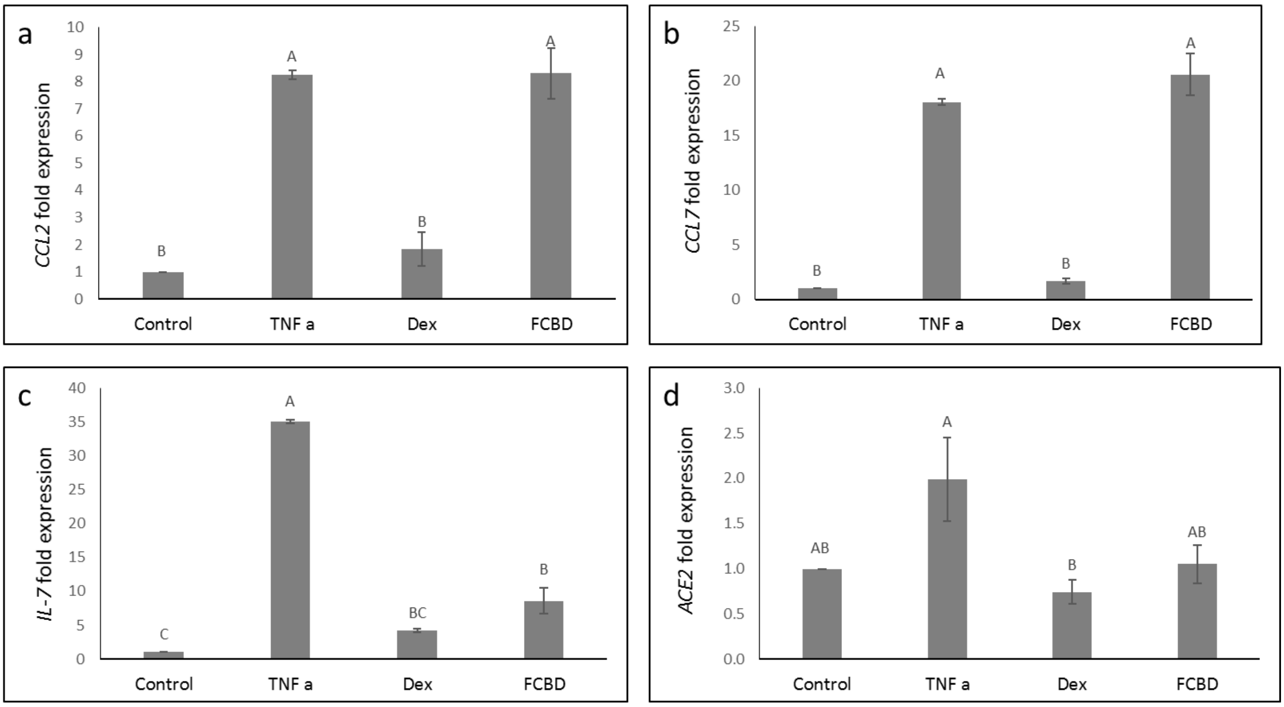

Supplementary Figure S5. Quantitative PCR (qPCR)-based determination of the mRNA steady state level of (a) *CCL2*, (b) *CCL7*, (c) *IL-7* or (d) *ACE2* genes in the A549 cell line, after treatment with TNFα (300 µg/mL) and F$_{CBD}$ (FCBD) at 7 µg/mL, or Dexamethasone (Dex) 4 µg/mL for 4 h, relative to the control. Gene transcription values were determined by qPCR as a ratio between the target gene and the reference gene (*HYPOXANTHINE PHOSPHORIBOSYLTRANSFERASE*1; *HPRT1*; geneID 3251). Values were calculated relative to the average expression of target genes in the treated cells versus control using the $2^{\Delta\Delta Ct}$ method. Control (0.7% v/v methanol) treatment served as the solvent (vehicle) control; TNFα indicates TNFα+solvent control treatment. Error bars indicate ± s.e.m. (n = 3). Bars labeled with different letters are significantly different from all combinations of pairs according to the Tukey-Kramer honest significant difference test (HSD; $P \leq 0.05$).

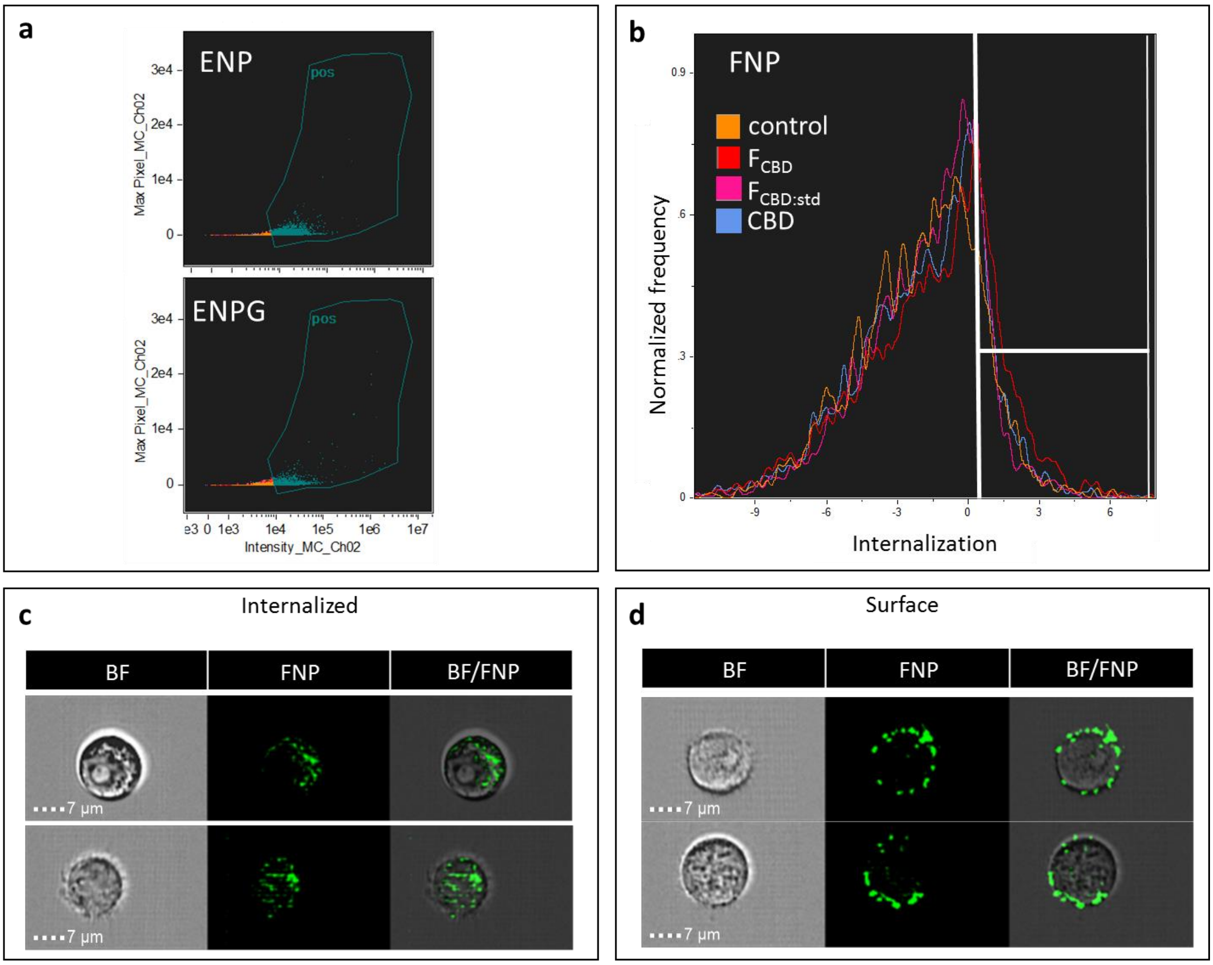

Supplementary Figure S6. (a, b) Examples of Imaging Flow Cytometry plots of macrophages. Macrophages (differentiated KG1 cells) were treated for 16 h with F_{CBD} at 7 µg/mL, $F_{CBD:std}$ at 7 µg/mL, CBD at 4.35 µg/mL and solvent (vehicle) control and then incubated for 4 h with silica beads (40 µg/mL). Silica beads included fluorescent-labeled silica 50-100 nm particles (FNP), fluorescent-labeled silica 30-70 nm particles (ENP) and IgG coated, fluorescent-labeled silica 30-70 nm particles (ENPG). At least 4,000 cells for each treatment were analyzed using Amnis IDEAS software and the distribution of the cell internalization scores were plotted (marked in white lines under the curves). (**a**) Examples for images of plots for cells positive (pos) for ENP/ENPG. (**b**) Example of images of plots for cells gated with FNP-internalization score higher than 0.33. **(c, d) Representative images of cells with internalized FNP beads (c) or beads on their surface (d), captured by Amnis ImageStreamX.** The first column shows brightfield (BF) images of the cells, the second column shows the fluorescent FNP beads only, and the third column shows the merged image (BF/FNP). Scale bar = 7 µm.
